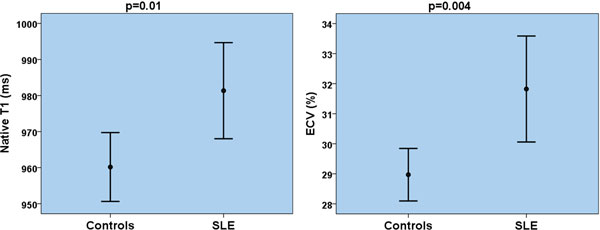# Diffuse myocardial fibrosis is subclinical and is associated with impaired myocardial deformation characteristics in systemic lupus erythematosus: a cardiovascular magnetic resonance study

**DOI:** 10.1186/1532-429X-16-S1-P307

**Published:** 2014-01-16

**Authors:** Ntobeko A Ntusi, Stefan K Piechnik, Jane M Francis, Vanessa M Ferreira, Paul M Matthews, Matthew D Robson, Paul B Wordsworth, Stefan Neubauer, Theodoros D Karamitsos

**Affiliations:** 1 Division of Cardiovascular Medicine, Radcliffe Department of Medicine, University of Oxford & John Radcliffe Hospital, Oxford, UK; 2 GlaxoSmithKline Clinical Imaging Centre, GlaxoSmithKline, London, UK; 3 Division of Brain Sciences, Department of Medicine, Imperial College, London, UK; 4 NIHR Oxford Musculoskeletal Biomedical Research Unit & Nuffield Department of Orthopaedics, Rheumatology and Musculoskeletal Sciences, University of Oxford & Nuffield Orthopaedic Centre & John Radcliffe Hospital, Oxford, UK

## Background

Systemic lupus erythematosus (SLE) is a systemic autoimmune disorder that commonly affects the heart, resulting in a 7 to 9 times greater incidence of cardiovascular disease (CVD) in SLE patients compared to healthy controls. Female patients with SLE between 35 and 44 years old have an incidence of myocardial infarction over 50 times greater than that observed in the Framingham cohort. Diffuse myocardial fibrosis can be detected non-invasively by extracellular volume (ECV) mapping based on pre- and postcontrast T1 measurements using cardiovascular magnetic resonance (CMR). We aimed to detect subclinical diffuse myocardial fibrosis in SLE using CMR T1 mapping.

## Methods

23 SLE patients (22 female, mean age 41 ± 9 years) and 23 matched controls (22 female, mean age 42 ± 9 years) without previously known cardiovascular disease underwent CMR at 1.5T. CMR evaluation included late gadolinium enhancement (LGE) [IV gadoterate meglumine at 0.15 mmol/kg], T1 mapping pre- and postcontrast, cine, tagging, and T2-weighted imaging.

## Results

Regional fibrosis on LGE imaging was found in 5 SLE patients (22%) compared to none of controls. Presence of diffuse myocardial fibrosis in SLE was confirmed by significantly higher precontrast T1 values (981 ± 31 vs. 960 ± 21 ms, p = 0.010), decreased postcontrast T1 values (445 ± 31 vs. 470 ± 24 ms, p = 0.005) and expansion of ECV (31.8 ± 4.1 vs. 28.9 ± 2.0 %, p = 0.004). Diffuse myocardial fibrosis was evident in SLE regardless of the presence of any regional fibrosis. Left ventricular volumes, mass and ejection fraction were similar between SLE patients and controls. However, peak systolic circumferential strain (-17.0 ± 1.6 vs. -19.3 ± 1.1, p < 0.001) and peak diastolic strain rate (79 ± 26 vs. 119 ± 15 s-1, p < 0.001) were impaired in SLE. Presence of diastolic dysfunction is SLE was further supported by larger left atrial diameters (31 ± 5 vs. 26 ± 4 mm, p < 0.001). Abnormal myocardial systolic strain and diastolic strain rate correlated with diffuse myocardial fibrosis indices. There was no evidence of myocardial edema in SLE.

## Conclusions

Cardiac involvement is common in SLE patients with no cardiovascular symptoms, and includes both focal and diffuse myocardial fibrosis, which is associated with impaired systolic and diastolic strain parameters. CMR is a robust non-invasive tool for the assessment of diffuse myocardial fibrosis and subclinical cardiac involvement in inflammatory heart disease.

## Funding

This study was funded by an investigator-led grant from GSK to Dr Theo Karamitsos. The authors gratefully acknowledge support from the National Institute for Health Research Oxford Biomedical Research Centre Programme. Prof. Stefan Neubauer also acknowledges support from the Oxford British Heart Foundation Centre for Research Excellence.

**Table 1:** Baseline characteristics and CMR findings

	Controls N = 23	SLE N = 23	P value
Female sex, n (%)	22 (92)	22 (92)	1.00

Age, years	42 ± 9	41 ± 9	0.68

Current smokers, n (%)	1 (4)	3 (13)	0.10

Hypertension, n (%)	0	0	-

Diabetes, n (%)	0	0	-

Hyperlipidaemia, n (%)	1 (4)	0	-

BMI, kg/m2	23 ± 3	28 ± 6	< 0.001

SLEDAI (median, IQR)	N/A	9 (7-14)	-

ESR, mm/hr (median, IQR)	N/A	6 (4-9)	-

CRP, mg/L (median, IQR)	1 (0-1)	3 (2-6)	< 0.001

Hemoglobin, g/L	13 ± 1	12 ± 1	0.37

Duration of SLE, years (median, IRQ)	N/A	9 (6-14)	-

Duration of DMARDs, years (median, IQR)	N/A	7 (4-10)	-

LVEDV indexed to BSA, ml/m2	79 ± 15	78 ± 13	0.91

LVESV indexed to BSA, ml/m2	21 ± 5	20 ± 6	0.43

LVEF, %	74 ± 5	72 ± 5	0.17

LV Mass indexed to BSA, g/m2	49 ± 10	46 ± 11	0.21

LA size, mm	26 ± 4	31 ± 5	< 0.001

Mid SA circumferential strain	-19.3 ± 1.1	-17.0 ± 1.6	< 0.001

Peak diastolic circumferential strain rate (s-1)	119 ± 15	79 ± 26	< 0.001

Presence of LGE (%)	0	5 (22)	-

Volume fraction of LGE > 2SD (%) STIR T2 Ratio	0	2.6 ± 0.2	-

STIR T2 Ratio	1.5 ± 0.1	1.6 ± 0.1	0.06

Continuous data are mean ± SD unless otherwise indicated. BMI, body mass index; CRP, C-reactive protein; DMARD, disease modifying anti-rheumatic drug; ESR, erythrocyte sedimentation rate; IQR, interquartile range; LA, left atrium; LGE, late gadolinium enhancement; LV, left ventricle/ventricular; LVEDV, left ventricular end-diastolic volume; LVEF, left ventricular ejection fraction; LVESV, left ventricular end-systolic volume; SA, short axis; SLEDAI, systemic lupus erythematosus disease activity index; SLE, systemic lupus erythematosus; STIR, short Tau inversion recovery

**Figure 1:**